# Global Changes in Asexual *Epichloë* Transcriptomes during the Early Stages, from Seed to Seedling, of Symbiotum Establishment

**DOI:** 10.3390/microorganisms9050991

**Published:** 2021-05-04

**Authors:** Inoka K. Hettiarachchige, Christy J. Vander Jagt, Ross C. Mann, Timothy I. Sawbridge, German C. Spangenberg, Kathryn M. Guthridge

**Affiliations:** 1 Agriculture Victoria, AgriBio, Centre for AgriBioscience, Bundoora, VIC 3083, Australia; inoka.hettiarachchige@agriculture.vic.gov.au (I.K.H.); christy.vanderjagt@agriculture.vic.gov.au (C.J.V.J.); ross.mann@agriculture.vic.gov.au (R.C.M.); tim.sawbridge@agriculture.vic.gov.au (T.I.S.); german.spangenberg@agriculture.vic.gov.au (G.C.S.); 2 School of Applied Systems Biology, La Trobe University, Bundoora, VIC 3086, Australia

**Keywords:** endophyte, transcriptome analysis, RNA sequencing, gene expression, alkaloid, defence response

## Abstract

Asexual *Epichloë* fungi are strictly seed-transmitted endophytic symbionts of cool-season grasses and spend their entire life cycle within the host plant. Endophyte infection can confer protective benefits to its host through the production of bioprotective compounds. Inversely, plants provide nourishment and shelter to the resident endophyte in return. Current understanding of the changes in global gene expression of asexual *Epichloë* endophytes during the early stages of host-endophyte symbiotum is limited. A time-course study using a deep RNA-sequencing approach was performed at six stages of germination, using seeds infected with one of three endophyte strains belonging to different representative taxa. Analysis of the most abundantly expressed endophyte genes identified that most were predicted to have a role in stress and defence responses. The number of differentially expressed genes observed at early time points was greater than those detected at later time points, suggesting an active transcriptional reprogramming of endophytes at the onset of seed germination. Gene ontology enrichment analysis revealed dynamic changes in global gene expression consistent with the developmental processes of symbiotic relationships. Expression of pathway genes for biosynthesis of key secondary metabolites was studied comprehensively and fuzzy clustering identified some unique expression patterns. Furthermore, comparisons of the transcriptomes from three endophyte strains in planta identified genes unique to each strain, including genes predicted to be associated with secondary metabolism. Findings from this study highlight the importance of better understanding the unique properties of individual endophyte strains and will serve as an excellent resource for future studies of host-endophyte interactions.

## 1. Introduction

Asexual *Epichloë* endophytes, previously classified as *Neotyphodium* (henceforth referred to as endophytes), of the family Clavicipitaceae, form symbiotic associations with cool-season grasses [1,2,3]. Most endophyte species form associations specific to individual host species, genera, or tribes in the Pööideae, a subfamily of the Poaceae [4,5]. Perennial ryegrass (*Lolium perenne* L.) is one of the most commonly utilised cool-season grass species in temperate pastoral agriculture, due to its favourable properties such as rapid growth and establishment as well as high forage quality [6]. The symbiotic interaction between Pööideae grasses and *Epichloë* endophytes has been studied most intensively because of its importance in agricultural pastoral systems [7].

Associations between *Epichloë* endophytic fungi and grasses are generally considered to be mutualistic, as both partners benefit, and the interaction occurs without inducing any pathological symptoms or injury to the host [8,9,10,11]. Endophyte infection can confer a variety of benefits including environmental stress tolerance (e.g., drought, cold, salt, and waterlogging), protection from both herbivory (e.g., livestock, birds, insects, nematodes), and disease through the production of bio-protective compounds [10,12,13]. Conversely, the major benefits to the endophyte include access to nutrients from the plant apoplastic space, shelter, and a mode of transmission via the seed [12,14].

Grass-endophyte associations produce a range of endophyte-derived bioactive alkaloids, such as peramine, ergot alkaloids, indole-diterpenes (lolitrems and epoxy-janthitrems), and lolines, which enhance the competitive ability of endophyte-infected grasses [15,16,17,18,19,20]. Of these alkaloids, peramine and the lolines are known to provide protection against invertebrates [21,22], while ergovaline and the lolitrems are detrimental to both grazing mammals and insects [23,24]. Epoxy-janthitrems are suggested to be tremorgenic in grazing mammals while involved in protecting the pasture against damage by insect pests [16,25]. The use of *Epichloë* endophytes is required for sustainable pasture production and persistence, particularly in areas where there is significant impact on pasture production by insect pests [26,27].

The life cycle of asexual *Epichloë* species is wholly confined within the host plant; endophyte hyphae remain alive and metabolically active throughout the life of the host grass [9,28,29]. In the seed, hyphae remain dormant until germination and endophyte colonization occurs via the apical meristem [30]. Infection of new tillers subsequently occurs through colonization of leaf primordia and axillary buds [26,31,32,33]. The endophyte exclusively colonizes above-ground tissues such as leaf primordia, leaf sheaths, and leaf blades; the distribution of hyphae within tillers exhibits a basal-to-apical concentration gradient [12]. Hyphae grow through the apoplastic spaces by intercalary hyphal extension and their growth is synchronized with the growth and development of the host plant [33,34,35]. To complete the life cycle, hyphae growing in developing inflorescences colonize the ovaries and ovules of the florets, infecting the developing embryo. The endophyte is then efficiently dispersed through host seeds to the next generation in a process termed vertical transmission [9,31,36,37].

Some aspects of grass-*Epichloë* interactions, such as the biosynthesis, accumulation and ecological consequences of the known secondary metabolites and enhanced stress tolerance are well characterised. However, in addition to these well-known major alkaloid classes, evidence suggests that there are other, as yet unexplored, endophytic compounds that benefit the symbiotum [13,38]. The involvement of yet undiscovered mechanisms such as endophyte-mediated changes in host defence chemistry, apart from the resistance provided by alkaloids has also been suggested [7]. Moreover, the basis for some aspects of the association, such as the effect of endophyte on other plant performance parameters and the molecular mechanisms that regulate the mutualistic interaction are still largely unknown [39,40,41,42]. A number of transcriptome studies (e.g., RNA-Seq, microarray, ESTs) have investigated numerous aspects of host-*Epichloë* interactions. This includes studies of transcriptome changes in the host caused by *Epichloë* infection [40,41], differential expression of genes in response to the presence of *Epichloë* species in several different host grass species [34,43,44,45], differential expression of plant and fungal genes in different plant tissues infected with endophyte [46] and transcriptomics analyses of mutants for certain genes versus wild type *E. festucae* in culture and in infected plants [42,47].

Transmission of endophyte from host seed to seedling, and establishment of a stable grass-endophyte association, is a critical stage in the endophyte lifecycle. However, so far, the focus has been primarily on other aspects of grass-endophyte interactions and an investigation of whole-transcriptome changes of endophytes in perennial ryegrass-asexual *Epichloë* symbioses has not been investigated. In this study, comprehensive RNA-Seq analysis of a time-course experiment was performed for three perennial ryegrass-endophyte symbioses, to gain a greater understanding of the transcriptional response of different endophytes in the transition from seed dormancy to colonization during seed germination and seedling growth. 

## 2. Materials and Methods

### 2.1. Plant Material and Seed Germination

Perennial ryegrass cultivar Alto seeds containing Standard Endophyte (SE), NEA11, and NEA12 were obtained from Barenbrug New Zealand, Christchurch, New Zealand. Properties of the endophyte strains selected for transcriptome analysis are summarised in Table 1. DNA was extracted from representative, random samples (88 samples) of 5-day old seedlings from each seed batch using the DNeasy Plant Mini Kit (QIAGEN, Hilden, Germany) and screened using DNA-based diagnostics to confirm the viability of endophyte.

**Figure 1:**
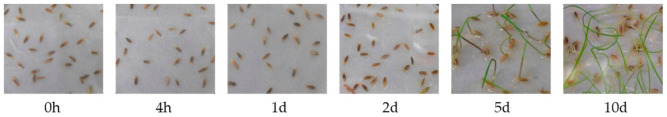
The six stages of seed germination used for analysis of the endophyte transcriptome in planta. Time points examined were 0 h, 4 h, 1 d, 2 d, 5 d, and 10 d.

Seeds were then sterilised with 5% NaOCl (sodium hypochlorite) with gentle shaking for 3 h. Seeds were washed thoroughly with sterile water and germinated on wet sterile filter papers in the dark for 2 days and in the light for a further 8 days (23 °C, 80 µMm^−2^s^−1^, 8–16 h photoperiod). During the germination process, 6 periods of germination were investigated, 0 hour (0 h), 4 hour (4 h), 1 day (1 d), 2 days (2 d), 5 days (5 d), and 10 days (10 d) (Figure 1). It is important to note that the time points used in this analysis were immediately after the 3.5-h sterilisation process. Twelve seeds at times 0 h, 4 h, 1 d, 2 d, and 6 seedlings at 5 d, 10 d per endophyte were harvested. Four biological replicates were prepared for each time point for each endophyte strain. 

### 2.2. RNA Extraction, Library Construction, and Sequencing

RNA was extracted using a CTAB-based method as previously described [50] with minor modifications.

Libraries were prepared using the Agilent SureSelect strand-specific RNA library preparation kit (Agilent Technologies, Santa Clara, CA, USA). RNA integrity was evaluated using an Agilent 2200 TapeStation (Agilent Technologies, Santa Clara, CA, USA) and each sample had an RIN^e^ value of above 7.0. Sequencing was performed on an Illumina Hiseq 3000 platform (http://www.illumina.com (accessed on 1 April 2021)) (Illumina Inc., San Diego, CA, USA).

### 2.3. Reference Genomes

Previously generated genome assemblies from C09, NEA11, and NEA12 strains [16,48] were used in this analysis. The C09 strain is genetically very similar to SE used in this study, and it was used as the reference genome for SE due to the availability of a high-quality reference genome [48]. A combination of reference gene sequences from *E. festucae* var. *lolii* C09 and *E. typhina* E8 was used for NEA11 due to its hybrid origin. Gene prediction for the reference genome assemblies was performed using the annotation program, AUGUSTUS (version 3.2, Göttingen, Germany) based on gene models for *Fusarium* *graminearum* [51]. Copies of the known lolitrem B and ergovaline genes were included as they were not predicted by AUGUSTUS. Functional annotation was done using Blast2go Pro (version 2.6.4, Valencia, Spain) [52].

### 2.4. Preprocessing and Mapping of Illumina Reads

Raw sequence reads were quality trimmed using the Gydle nuclear program (http://www.gydle.com (accessed on 1 April 2021)) (Gydle Inc., Quebec, QC, Canada), such that all reads ≥50 bases long were retained using a base quality cut-off value of 20. All high-quality reads for a given endophyte strain were mapped separately to the corresponding endophyte reference sequences using Gydle nuclear program. The filtered reads were also mapped to a database of perennial ryegrass [53].

The number of reads mapped to individual genes in the reference genome was used to generate count matrices for downstream analysis. Count matrices were normalized for library size using Bioconductor R package, DESeq (version 1.14.0, Heidelberg, Germany) [54]. Sample relations were plotted using a multidimensional scaling plot (MDS) generated with the Bioconductor R package, edgeR, Victoria, Australia) [55].

### 2.5. Identification of the Most Highly Expressed Genes—The Top 50

The most highly expressed genes were identified by sorting according to their expression levels, considering the total number of reads mapped to endophyte genes at all 6 time points of analysis. The top 50 annotated genes of NEA11 identified homologous genes from both progenitor species, *E. festucae* and *E. typhina*. However, as predominantly the same endophyte-derived reads were mapped to the same homologous genes from both progenitors, genes with the highest read counts were considered in this analysis.

### 2.6. Differential Gene Expression Analysis

Genes with ≥10 normalised reads for any of the 4 biological replicates and for any of the 6 seed germination stages were considered expressed. Differential gene expression analysis was performed using edgeR. Differentially expressed genes (DEGs) were identified based on comparisons between the expression in one time point compared to the average expression of that particular gene across all time points. Fold change ≥ 2 and *p* ≤ 0.01 were set as the threshold for the selection of DEGs.

DEGs were annotated using Blast2GO Pro. BlastX was run though QBlast against the NCBI nr-database, followed by mapping and annotation using default parameter values. A comparison against the InterPro domain database was used to increase the number of annotated sequences, using InterProScan. Blast2go software was also used to annotate the DEGs’ major Gene Ontology (GO) into three categories: molecular functions, biological processes, and cellular components.

### 2.7. Enrichment Analysis

Enrichment analyses of upregulated genes at each time point were run within Blast2GO using Fisher’s Exact Test. This analysis detected over-represented functional GO terms within the GO categories; cellular component, biological processes, and molecular functions with statistical significance [false discovery rate (FDR) < 0.05]. To achieve this, DEGs at each time point were compared with the remaining non-differentially expressed genes (used as reference transcriptome). GO terms of enrichment test outcomes were then sorted by (FDR) < 0.05 to identify the most specific GO terms. REVIGO software (Reduce and Visualize Gene Ontology) (http://revigo.irb.hr (accessed on 1 April 2021)) [56] was used to refine and visualize the similarity between the enriched terms.

### 2.8. Clustering

Fuzzy clustering of genes with similar expression patterns across the six seed germination stages was performed using the R package, Mfuzz [57]. Only genes with significant differences in expression, in at least one time point [log_2_FC2 (log_2_FoldChange), *p* ≤ 0.01), were used as input for the clustering. Soft clustering was then performed using the fuzzy c-means algorithm with the following parameters: c (number of clusters) = 12, m (fuzzifier) = 1.25. Genes were assigned to clusters of given expression patterns based on having a membership value >0.7.

### 2.9. Identification of Unique Genes of Each Endophyte Strain

The filtered sequencing reads of all three endophyte strains—SE, NEA11, and NEA12—were mapped separately to reference gene sequences of each endophyte strain. For example, RNAseq reads of SE were mapped to both NEA11 and NEA12 reference genomes in addition to the SE reference genome (C09). Identification of unique genes for each endophyte strain was based on the normalised number of reads; genes with more than 25 reads for at least one time point for that particular taxonomic group compared to having not more than 5 reads for any time point in the other two taxonomic groups were considered as unique.

## 3. Results and Discussion

### 3.1. Library Preparation and Mapping Reads to Endophyte Genome

In this study, the mixed transcriptomes of endophyte and host plant were sequenced. Libraries were prepared from RNA extracted from Alto-SE, Alto-NEA11, and Alto-NEA12 seeds and seedlings at six time points following confirmation of endophyte presence and viability using DNA-based diagnostics. Percentages of viable endophyte in seed were 94.3%, 91.4%, and 95.7% for Alto-SE, Alto-NEA11, and Alto-NEA12 respectively.

The number of raw reads generated for each biological replicate ranged from 4 to 70 million and more than 10 million reads were generated by 47/72 (65.28%) libraries. The biological replicates of different time points were used to produce multidimensional scaling (MDS) plots to check for variations among replicates (Figure S1). Clear differentiation of biological replicates at different time points and low variability among replicates at each time point were observed.

**Figure 2:**
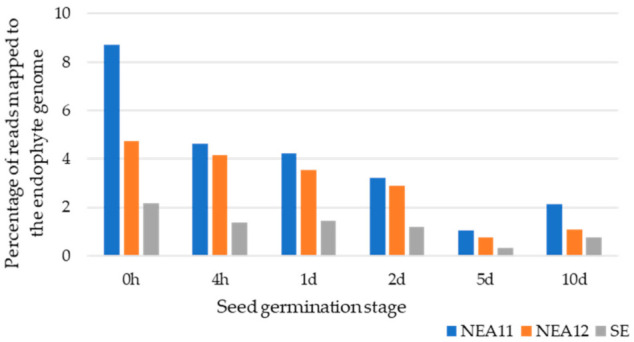
Percentages of total number of reads mapped to endophyte reference genomes for SE, NEA11, and NEA12 at different time points of seed germination during early stages of symbiotum establishment.

As expected, libraries largely comprised of perennial ryegrass sequences (91.28–99.66%) at the time points examined, and in all but one instance the percentage of endophyte reads mapped was below 5%, reflecting the low abundance of endophyte biomass in infected seed and plant material (Figure 2). Consistent with this study, low endophyte to host biomass ratios have been observed in other studies of grass-endophyte interactions [45]. The proportions in transcript abundance observed for the two organisms are largely due to differences in genome sizes and biomass of the host plant and endophyte. The estimated genome size of perennial ryegrass is around 2.6 Gb [58] while the genome sizes of the asexual haploid *Epichloë* endophytes and hybrid anamorphs are 29 ± 4 Mb (*E. festucae* isolate E189 and *E. typhina* E8) and 55 ± 7 Mb (*E. festucae* var. *lolii* × *E. typhina* Lp1), respectively [59]. Most recently, the *E. festucae* strain Fl1 genome was estimated at 35 Mb [60]. 

In general, the percentage of reads that mapped to an endophyte genome declined from the beginning of seed germination (0 h) to 5d. Then, an increase in reads mapped was observed at 10d. At the initial stage of symbiotum establishment (0 h), the proportion of endophyte cells present in the seed is high compared to later time points where shoots and roots have developed. Thus, a decrease in the proportion of reads that map to an endophyte genome with time is likely due to changes in the relative biomass of the two partners in the association. The highest and lowest percentage of reads mapped to endophyte reference genome were observed for NEA11 (possibly due to its heteroploidy) and SE respectively for all time points.

### 3.2. Insights into the Most Highly Expressed Genes during Early Stages of Symbiotum Establishment—The Top 50

Closer analysis of the most highly expressed genes provides insights into the processes to which the resident endophyte expends its energy in the first few days of symbiotum establishment. Of the three strains investigated, the percentage of total reads that mapped to the top 50 annotated genes of SE, NEA11, and NEA12 was 17.32%, 9.67%, and 12.97% respectively. Of these 50 genes, SE and NEA11 have 28 common genes, likely due to each having an *E. festucae* progenitor.

#### 3.2.1. The Most Abundant Endophyte Genes Expressed by SE, NEA11, and NEA12

**Figure 3:**
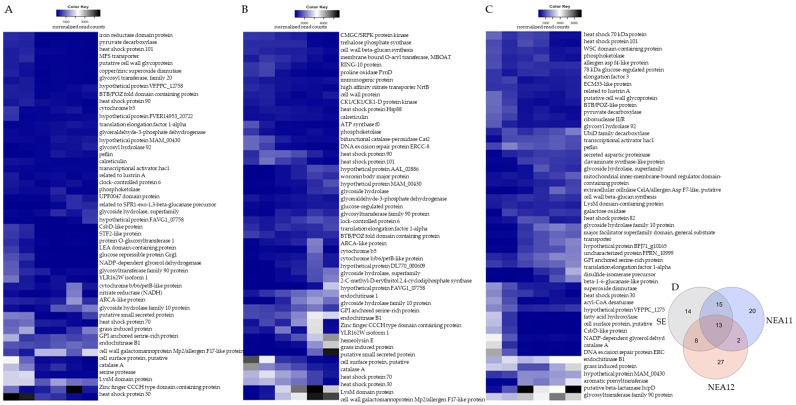
Heat map of the top 50 expressed genes with annotations (**A**) SE, (**B**) NEA11, and (**C**) NEA12. The colour key shown on the top right of each heat map represents the normalized read counts. (**D**) Venn diagram showing the common and unique sets of the most highly expressed genes of SE, NEA11, and NEA12 during early stages of symbiotum establishment (Venn diagram was created using the InteractiVenn online tool [84]).

The top 50 most abundantly expressed endophyte genes for SE, NEA11 and NEA12 are shown in a heatmap (Figure 3) and their top BLAST hits are available in Table S1. Most of these genes have predicted functions related to stress and defence responses, carbohydrate metabolism, and transport. 

The most abundant genes expressed by SE, NEA11, and NEA12 show homology to heat shock protein 30 (Hsp30), gene coding LysM domain protein, and putative β-lactamase HcpD, respectively.

Both plant and pathogen experience stress due to the activities of degradative enzymes, different reactive radicals, and extreme pH as a consequence of pathogen invasion of host tissue and HSPs are synthesised as a protective response to maintain cellular integrity [61]. In general, HSPs participate in morphogenesis of fungi and play an important role in replication, transcription and post-transcriptional processes; translation and post-translational processes, activation of signalling pathways and scavenging ROS (reactive oxygen species) [62,63]. HSPs are also involved in maintaining cellular homeostasis through correct folding of stress-accumulated misfolded proteins, preventing unfolded proteins from aggregation, and promoting selective degradation of misfolded or denatured proteins [64]. In addition to *Hsp30*, other HSP genes including *Hsp70*, *Hsp90,* and *Hsp101* were also among the 50 most abundantly expressed genes in the three endophyte transcriptomes.

In phytopathogenic fungi, LysM effector proteins have distinct roles in protecting fungi by binding to chitin, thus suppressing the chitin-triggered host defence responses and protecting fungal hyphae from degradation by chitinases [46,65]. LysM proteins may have a similar role in maintaining plant-fungi symbiotic interactions. This is further supported by their high level of expression in *Epichloë*-infected plant tissues in previous studies [34,46].

Many genes predicted to code for proteins that are highly similar to β-lactamases have been identified in fungi, however, functions of only a few gene products have been confirmed so far compared to well-characterised bacterial β-lactamases [66]. It is hypothesised that hydrolytic β-lactamases are involved in degradation of xenobiotic lactam compounds produced by plants or other microbes [66]. 

#### 3.2.2. The Most Abundant Endophyte Genes Common to SE, NEA11, and NEA12

Analysis of the most abundant genes common to all three strains identified 13 genes. Most of these genes, except hypothetical protein MAM_00430 and cell surface protein, have already been characterised in *Epichloë* or other fungal species confirming the validity of this study (Table 2).

Catalase A is one of the most important antioxidant enzymes. Significantly higher levels of expression for the gene encoding catalase; *catA*, together with other genes associated with stress responses, has been observed for ovaries over vegetative pseudostems in *E. coenophiala*-infected tall fescue [41]. It has been suggested by others that endophytes may upregulate ROS scavenging enzymes, such as catalase A, to remove autoregulatory inhibition of endophyte growth, which then allows the endophyte to colonize differentiating plant tissues [41].

Higher expression of *E. festucae* GPI-anchored proteins in planta compared to in culture has also been observed in previous studies, suggesting the importance of these genes in host-endophyte interactions [67]. RhgA functions as a putative GPI-anchored cell wall protein in *E. festucae* and is involved in maintaining normal hyphal growth during infection [68]. Interestingly, *rghA* was found among the 50 highest expressed genes in both SE and NEA12 symbiota.

Eukaryotic translation elongation factor 1-alpha, TefA, is one of the most abundant proteins in the cell [69]. The gene *tefA* has been identified as one of the most abundantly expressed *E. festucae* transcripts in *Epichloë*-infected plant tissues in other studies [46].

Carbohydrate-active enzymes (CAZymes) were also among the top 50 genes common to the three endophyte strains. Two genes important for CAZyme gene expression during host-*E. festucae* interactions, *velA,* and *laeA,* have been identified in a recent study [42].

The function of hypothetical protein MAM_00430 is unknown, although it is among the top 50 expressed genes of all three endophyte strains. Putative cell surface protein coded by another highly expressed gene identified as common to all three strains has also not been characterised in *Epichloë*. The high expression levels of these two genes warrant further study to characterise and confirm their roles in host-*Epichloë* interactions.

#### 3.2.3. Other Highly Expressed Endophyte Genes

Twenty-five genes were identified as common to at least two endophyte strains, including well-characterised genes coding for LysM domain protein, cell wall galactomannoprotein Mp2/allergen F17-like protein, putative cell wall glycoprotein, cell wall beta-glucan synthesis, Zinc finger CCCH type domain-containing protein, putative small secreted protein, clock-controlled protein 6, peflin, ARCA-like protein, BTB/POZ fold domain-containing protein, glycosyl hydrolase 92, cytochrome b/b6/petB-like protein, glyceraldehyde-3-phosphate dehydrogenase, Hsp70, Hsp90, DNA excision repair protein ERCC-8, Cytochrome b5, NADP-dependent glycerol dehydrogenase, related to lustrin A, CsbD-like protein, transcriptional activator hac1, calreticulin and less-characterised gene coding for YLR162W, hypothetical protein VFPPC_12758 and hypothetical protein FAVG1_0775.

Some of these genes such as cell wall proteins, small secreted proteins, glyceraldehyde-3-phosphate dehydrogenase, and clock controlled proteins have been described in previous studies as related to the host-*Epichloë* symbiotum. 

Cell wall proteins have many different roles, including defending the fungus from environmental stresses in the host environment whilst maintaining the structural integrity of the cell wall [73,79]. Cell wall proteins are also involved in adhesion, evident from the observation that the endophyte cell wall attaches to the host cell wall in *L. perenne*-*E. festucae* var. *lolii* interactions [68]. In addition to the GPI-anchored proteins described above, other cell wall protein-related genes such as cell wall β-glucan synthesis, putative cell wall glycoprotein, and cell wall galactomannoprotein Mp2/allergen F17-like protein were among the highest expressed genes in this study.

Secreted proteins are thought to have various roles in host-endophyte interactions, including communication between the two partners in the interaction [68]. While functions of most of these genes are unknown, some are characterized as small cysteine-rich proteins with potential roles in the host-endophyte symbiosis [34]. High expression of endophyte transcripts for secreted proteins was observed in the analysis of *E. festucae* and creeping red fescue (*Festuca rubra* L. subsp. *rubra*) associations [34]. Effector proteins, important for colonization of phytopathogens and endosymbionts, are also characterized as small-secreted proteins [34,46]. A gene encoding a small-secreted protein similar to cerato-platanins is overexpressed in choke stroma tissue of *E. festucae*-infected *F. rubra* [46]. Cerato-platanins are considered to be involved in loosening cell walls of both partners, fungus, and plant, facilitating hyphal penetration into the host tissue as well as the release of nutrients from plant cells [46,80]. Moreover, there is some evidence that secreted proteins have a role in host specialization of *Epichloë* spp. [46].

In concordance with previous studies in which glyceraldehyde-3-phosphate dehydrogenase (GAPDH) was among the most abundant *E. festucae* transcripts in asymptomatic inflorescence tissues of *E. festucae*-infected *F. rubra* [46], this gene was also very highly expressed by the endophyte species examined in this study. Glyceraldehyde-3-phosphate dehydrogenase is a moonlighting protein which plays a role in the virulence of pathogenic fungi [75].

Clock-controlled proteins have been identified as highly expressed in both asymptomatic inflorescence tissues and choke stroma tissues of *E. festucae*-infected *F. rubra* [46]. 

Genes with unknown roles in the plant-endophyte symbiosis were also identified among the top 50 expressed genes of at least two endophyte strains; hypothetical protein VFPPC_12758; YLR162W isoform 1; CsbD-like protein; and hypothetical protein FAVG1_07758. YLR162W is particularly interesting, as this gene is believed to play an important role in the response to antimicrobial peptides [81]. CsbD is a general stress response protein with an unknown function [82,83]. This gene has been identified among highly expressed genes in fungi such as *A. flavus* NRRL 35739 [82]. None of these genes have been characterized in *Epichloë* yet, however, the high expression levels of these genes in at least two endophyte strains representing different taxa suggest important roles during symbiosis and warrant further investigation.

### 3.3. Identification of Differentially Expressed Genes

**Figure 4:**
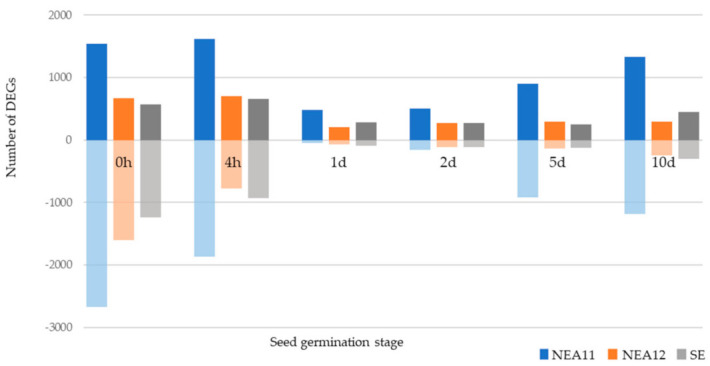
Number of DEGs of SE, NEA11, and NEA12 at different stages of seed germination during early stages of symbiotum establishment. The numbers of upregulated genes are shown in darker colour and downregulated in lighter colour.

A total of 3471, 6811, and 3161 genes were differentially expressed at one or more of the six time points for SE, NEA11, and NEA12, respectively (Figure 4). This represented a percentage of 59.93%, 42.61%, and 42.15% of all the genes expressed in this study.

For all three endophyte strains, the number of DEGs at the first two time points, 0 h and 4 h, were higher than the other four time points. This suggests that the largest changes in endophyte gene expression occurred at the onset of seed germination when dormant hyphae present in dry seeds enter a more active stage with seed imbibition. The number of downregulated genes were higher than upregulated genes at 0 h and 4 h in all three strains. From 1d, a higher number of upregulated genes were observed than downregulated genes in all strains at all time points, with the exception of NEA11 at 5d. Furthermore, all three strains have their highest number of upregulated genes at 4 h; conversely, all three strains showed the highest number of downregulated genes at 0 h. The number of DEGs was lowest at 1d in all three strains. For NEA11, a sharp increase in DEGs is observed from 2d indicating considerable changes in endophyte metabolism. SE and NEA12 show a more gradual increase in DEGs from 5d and 2d onwards, respectively.

### 3.4. Gene Ontology (GO) Functional Enrichment Analysis

To gain an understanding of the interaction between the host plant and endophyte during the early stages of symbiotum establishment, the biological importance of the DEGs at different time points was further investigated. GO annotation was used to identify the functional categories of upregulated genes of SE and is provided in Figure S2.

Enrichment analysis was performed on the DEG sets to determine the major GO (Biological Process) categories of genes upregulated at different time points for SE, NEA11, and NEA12. The full lists of enriched GO terms are presented in Table S2. 

*E. festucae* var. *lolii* strain SE, which is a strain of Standard Toxic wildtype endophyte, also known as “common endophyte”, was chosen for a detailed study. Standard Toxic strains are commonly observed in natural populations of ryegrass and synthesize three of the five important known secondary metabolites (peramine, ergovaline, and lolitrem B) [49,85]. Standard Toxic strains have also been used in many studies to investigate the effects of endophyte on perennial ryegrass-host performance [86,87]. 

**Figure 5:**
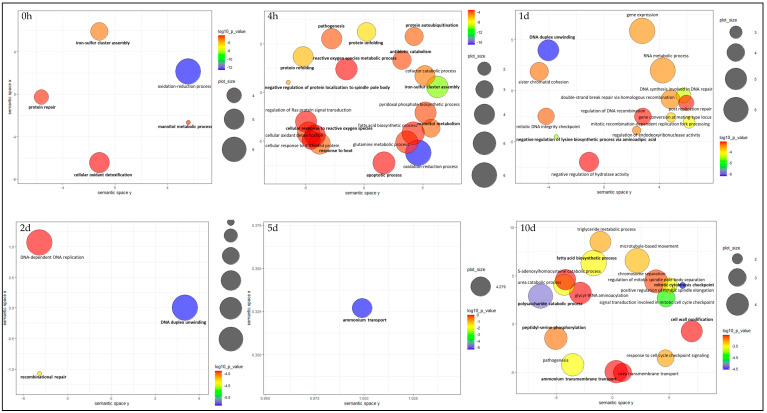
Enrichment analyses of GO terms related to upregulated biological processes of SE at six different stages of symbiotum establishment; summarized and visualized using REVIGO. Circles indicate GO terms and are plotted according to semantic similarities to other GO terms. Circle size is proportional to the frequency of the GO term, while colour indicates the log10 *P*-value (red higher, blue lower). Adjoining circles are most closely related. Cluster representatives are given in bold black font.

It is expected that changes in host development trigger the initiation of each stage of endophyte hyphal development [35]. Accordingly, in this study, the changes in global gene expression of SE observed are consistent with developmental processes of a symbiotic relationship. A general shift of main enriched GO terms at three stages (0 h and 4 h, 1d and 2d, 5d and 10d) was observed as shown in Figure 5. 

#### 3.4.1. 0 h and 4 h

At the beginning of the symbiotum establishment stage (0 h and 4 h), especially processes related to establishment of normal symbiotic interactions such as “iron metabolism”, “mannitol metabolism”, “cellular oxidant detoxification”, “response to heat”, “pathogenicity”, “reactive oxygen species metabolic process”, “apoptotic process”, and “protein folding” were enriched. 

Although *Epichloë* are endosymbionts, the initial phases of their interaction with the host do not seem to differ fundamentally from pathogenic fungi. Adaptations to access the host plant interior, utilize carbon and nitrogen compounds from the apoplastic space, and inactivation of potential defence responses by the host plant are essential for the functioning of symbiosis between host plant and endophyte [34,88].

In the initial stages of fungal colonization, the host plant produces ROS including superoxide, hydroperoxyl radicals, hydrogen peroxide, and hydroxyl radicals as just one of the earliest active defence responses [89,90]. Imbalance between ROS production and detoxification can lead to oxidative stress; oxidative damage to proteins, nucleic acids, and membranes; and ultimately cell death (apoptosis) [89,90]. Therefore, detoxification of ROS is vital for the maintenance of cellular integrity [89]. Further supporting these observations of high ROS production, the genes for catalase and SOD, which detoxifies ROS, were identified as highly expressed (in the top 50) genes in this study. Higher Cu/Zn-SOD levels in planta than in culture for *E. festucae* var. *lolii* have also been observed in other studies [91].

*Epichloë* endophytes and other fungi generate ROS by themselves as part of establishing a successful interaction with the plant, as has been shown in associations between *E. festucae* and *L. perenne* [92,93]. Similar to pathogenic fungi, Clavicipitacean endophytes also use ROS to denature plant cell membranes and to enhance leakage of nutrients from host cells into apoplastic spaces, making them available for fungal hyphae inhabiting the intercellular spaces [92,94].

Mannitol metabolism is another significantly enriched process at the initial time points of symbiotum establishment. Mannitol is one of the most common storage, and the most widely recognized, polyol in biological systems including filamentous fungi [95]. Mannitol has been shown to have antioxidant activity and therefore is a significant factor in pathogenicity [94,95]. Fungal pathogens secrete mannitol to quench ROS that mediate plant defences [96]. For example, tall fescue (*F. arundinacea*) grass tissues infected by *E. coenophiala* have higher concentrations of mannitol and other antioxidant fungal carbohydrates [90].

Metals act as cofactors in a multitude of enzymes which play important roles during growth and infection processes [97]. According to gene set enrichment analysis, iron seems to be a major component of maintaining a stable symbiotic interaction at the very beginning of symbiotum establishment. Pathogens compete with their host for iron and have developed different mechanisms for iron uptake such as from ferritin or via siderophores [98]. *E. festucae* use extracellular siderophores to obtain iron from the host plant [99]. Iron-sulfur (Fe–S) clusters are involved in essential cell functions including energy production, metabolic conversions, DNA maintenance, gene expression regulation, protein translation, and the antiviral response [100]. Furthermore, Fe–S clusters play important roles in iron homeostasis mechanisms, which is very important for symbiotic maintenance as loss of iron homeostasis leads to generation of ROS [101].

“Response to heat” is another enriched GO term which includes the activity of HSPs described above. Endophytes experience protein damage under stress conditions and “protein unfolding and refolding” were also among the enriched terms. 

#### 3.4.2. Day 1 and 2

The most highly enriched GO terms at day 1 and 2 were associated with cellular responses to DNA damage such as “chromosome organisation” and “cell division and recombination”. Further supporting this observation, genes for DNA repair proteins such as ERCC-8 and RAD5 were identified among the highest expressed endophyte genes in this study (top 100). Furthermore, protein kinases activated by DNA damage are also highly expressed in all three strains. Oxidative damage of endophyte DNA, caused by plant-produced defensive ROS during the infection process, includes modified nucleotide bases or single-strand breaks, chemical base changes, structural alterations, single- and double-strand breaks, and cross-linkage [102,103]. DNA double-strand break is a particularly dangerous damage and needs to be repaired for the cell to survive [104]. Several repair mechanisms, such as the base-excision repair (BER) pathway, double-strand break (DSB) repair pathways, nucleotide-excision repair (NER), and mismatch repair (MMR) play a key role in the repair of oxidative DNA damages caused by ROS in yeast and mammalian cells [102].

#### 3.4.3. Day 5 and 10

Day 5 and 10, where rapid growth of seedlings can be observed, “mitotic cytokinesis checkpoint”, “polysaccharide catabolism”, “triglyceride metabolism”, “ammonium transmembrane transport”, “peptidyl-serine phosphorylation”, “pathogenesis”, “response to cell cycle checkpoint signalling” and “cell wall modification” were among enriched GO categories. 

Regulation of host and symbiont cell cycles is very important to maintain a healthy symbiotic association [105]. This is particularly important for *Epichloë* endophytes, as their colonization is synchronized with the growth of the host plant [35]. So, the fact that mitotic cell cycle-related biological processes were enriched towards the later time points of analysis is particularly noticeable.

During seed germination, *E. festucae* grows between undifferentiated plant cells and colonizes newly developing leaves [106,107]. Furthermore, rapid colonization of endophytes in young plant tissues has been observed compared with the mature parts of the plant [41]. These observations suggest rapid growth of endophytes at the early stages of symbiotum establishment. Transporters were identified as playing a major role at this stage of seed germination and, more specifically, transporters required for nitrogen utilization, such as urea and ammonium, were identified among enriched GO terms. Fungal pathogens can experience nitrogen limitation and the importance of ammonium transporters during host infection has been reported in other studies [108].

Carbohydrate and lipid metabolism were also enriched at these stages. Lipid and sugar metabolism in fungi generates energy for survival, maintains turgor pressure, and facilitates melanin synthesis [109]. For example, trehalose is important for the survival and virulence of pathogenic fungi and also acts as a free radical scavenger under oxidative stress conditions [110,111]. The involvement of trehalose to regulate turgor of hyphae of *E. coenophiala*, which supports colonization of host tissues, was suggested based on the role of trehalose during previous plant-fungal pathogen interaction studies [41]. Furthermore, breaking down host polysaccharides and plant surface polymers is required to penetrate the host successfully as explained above [112]. Pathways involved in triglyceride metabolism and phospholipid homeostasis have also been shown to contribute to successful host infection [113].

Finally, and as described above, cell wall modifications are another enriched process that are essential for proper functioning of host-endophyte interactions.

### 3.5. Gene Expression Profiles of Known Alkaloid Biosynthesis Genes

Endophyte-derived alkaloids enhance survival of the host plant and are therefore an important aspect of the evolution of the host-endophyte interaction. Genes for the biosynthesis of the five main bioprotective alkaloids, lolitrem B, epoxy-janthitrems, ergovaline, peramine and the lolines and have now been identified [15,16,17,18,19,20]. Of those, only peramine is encoded by a single gene while the genes for the other secondary metabolite pathways are found in clusters in endophyte genomes.

Most genes involved in alkaloid biosynthesis were differentially expressed and in general, downregulated at initial time points and upregulated at later time points (Figure S3).

**Figure 6:**
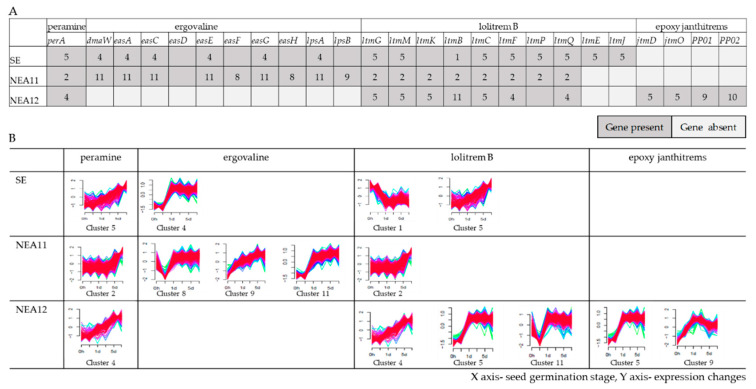
(**A**) Cluster numbers for secondary metabolism pathway genes of SE, NEA11, and NEA12. (**B**) Expression patterns of secondary metabolism pathway genes of SE, NEA11, and NEA12 identified across the time points of seed germination during early stages of symbiotum establishment by fuzzy clustering. Some pathway genes are not shown in this figure because their expression did not meet the filter threshold (significant differences in expression in at least one time point and log_2_FC2, *p* ≤ 0.01).

Cluster analysis of DEGs was performed to determine groups of genes with similar expression trends over the time course of seed germination (Figure S4). Of them, the expression patterns of pathway genes for five key secondary metabolites were investigated in detail, revealing that genes involved in alkaloid biosynthesis are present in few specific clusters (Figure 6A) and pathway genes belonging to a particular alkaloid have similar expression patterns (Figure 6B). For example, in the endophyte strain SE, the *perA* gene is present in cluster 5 and all pathway genes for ergovaline are present in cluster 4 (note: there is no cluster that contained *easD*, *easF,* and *easH* as they did not meet the filter threshold used for this analysis). Moreover, the genes responsible for lolitrem B biosynthesis which are arranged in a biosynthetic gene cluster in the *Epichloë* genome [114] showed similar expression profiles (cluster 5) except *ltmB* (cluster 1) in SE. This is consistent with the frequent observation of co-expression of physically linked genes in eukaryotes [115]. 

Biosynthesis genes for some alkaloids showed similar expression patterns in different endophyte strains. For instance, the *perA* gene has a similar expression pattern in SE, NEA11, and NEA12. Interestingly, although the full-length *perA* gene is not present in NEA12, expression of the truncated *perA* gene still followed a similar expression pattern [47]. 

Another interesting observation of this analysis is the identification of common expression patterns of different alkaloids. For example, most lolitrem B biosynthesis pathway genes from each of the three endophyte strains have a similar expression pattern to the *perA* gene. Interestingly, a positive correlation between expression level of peramine and lolitrem B has been observed in a study of perennial ryegrass-*E. festucae* var. *lolii* interaction [116].

Furthermore, another unique gene expression pattern for alkaloid biosynthesis genes common to all three endophyte strains was identified: lolitrem B biosynthesis genes (*ltmG*, *ltmM*, *ltmK,* and *ltmC*) of NEA12 (cluster 5), epoxy-janthitrem biosynthesis genes (*jtmD* and *jtmO*) of NEA12 (cluster 5), ergovaline pathway genes of SE (cluster 4) and NEA11 (*dmaW*, *easA*, *easC*, *easE*, *easG,* and *lpsA*- cluster 11) showed similar expression patterns. 

### 3.6. Identification of Unique Genes of Each Endophyte Strain 

The number of unique genes identified for SE, NEA11, and NEA12 was 2, 65, and 20 respectively. 

#### 3.6.1. Comparative Analysis Between SE and NEA12

Only two genes, *ltmE* and l*tmJ*, were identified as unique to SE, most probably due to having a common *E. festucae* progenitor for both SE and NEA11 [48]. Both NEA11 and NEA12 do not produce lolitrem B due to a lack of those two genes. 

Therefore, a comparison of genes expressed by SE and NEA12 was performed and 28 genes unique to SE were identified. Of them, 11 genes are known to be related to alkaloid biosynthesis: *ltmE*, *ltmJ,* and all pathway genes for ergovaline biosynthesis except *easF* (due to having less than 25 reads). Most of the other genes code for hypothetical proteins (Table S3). 

Interestingly, an NRPS (Ef_C09_g11720) was identified as unique to SE. NRPSs are large multifunctional proteins that synthesise a broad spectrum of bioactive compounds. More than 12 NRPS genes have been identified in different *Epichloë* spp., including the *perA* gene [117]. A non-ribosomal peptide synthetase A gene, *vlmS* from *Lecanicillium* sp. HF627 (GenBank accession: AB862312.1), was identified as the most similar homologue for this gene, based on blast analysis of the Genbank database. In *Lecanicillium* sp., *vlmS* is clustered with another three genes; fatty acid hydroxylase (*vlmA*), thioesterase (*vlmB*), and AMP-dependent ligase (*vlmC*) and this gene cluster is responsible for biosynthesis of verlamelins [118]. Verlamelins are cyclic lipopeptides with antifungal activity against plant pathogenic fungi such as *Magnaporthe oryzae* which causes a severe foliar disease of perennial ryegrass, gray leaf spot [118,119,120]. This NRPS gene coding verlamelin has also been overexpressed in the asymptomatic inflorescence tissue compared to choke stroma tissues of *E. festucae*-infected *F. rubra* [46]. Interestingly, Ef_C09_g11718, another gene identified as unique to SE from the comparison with NEA12, codes for putative AMP-dependent ligase/synthetase (*vlmC*). Both Ef_C09_g11720 and Ef_C09_g11718 are present in the same contig while a gene corresponding to *vlmB* (Ef_C09_g11719) is located between Ef_C09_g11718 and Ef_C09_g11720, however, this gene showed negligible expression. These three genes are present in close proximity to the ergot alkaloid biosynthetic gene cluster in the corresponding genomic contig. A gene with homology to *vlmA* (Ef_C09_g04986) is present in a different contig. 

Antifungal activity of SE has been observed against many pathogenic fungi and the compounds responsible for this are still unknown [13]. Hence, these genes warrant further investigation as verlamelin has a strong and broad-spectrum antifungal activity which may contribute to the bioprotection of the host.

#### 3.6.2. Genes Unique to NEA11

The number of genes identified as unique to NEA11 in this study was 65. Of the genes that had matches to known genes present in the NCBI databases, most of them have roles related to endophytic and or pathogenic behaviour. There were six hypothetical proteins and seven genes with no annotations. Top blast hits of all these genes are provided in Table S4.

Many CAZymes including glycoside hydrolase family 33 protein (GH33), glycosyl hydrolase family 92 protein, and 2 glycosyl transferase (GT) family proteins (17 and 90), as well as a putative beta-glucosidase (*btgE*) were identified. Furthermore, genes related to esterases, which are important for penetration of the host, were also among the genes unique to NEA11. Interestingly, there were another nine protein kinase-associated genes. Protein kinases regulate various cellular processes and have very important roles in fungal pathogenicity on plants [121].

Genes associated with secondary metabolite production such as cytochrome P450 and NRPS were also identified as unique to NEA11. Cytochrome P450s play important roles in ergosterol synthesis, virulence, and production of a range of secondary metabolites [122].

#### 3.6.3. Genes Unique to NEA12

Twenty genes were identified as unique to NEA12 and top blast hits are given in Table S5. Three of them, NEA12_g01210 (*jtm01*), NEA12_g01211 (*jtm02*), and NEA12_g01213 (*PP03*) (transposase with a MULE domain) are involved in epoxy-janthitrem biosynthesis [16]. A very high expression of *jtmO* and *jtmD* was observed for NEA12 compared to no or negligible expression level in SE and NEA11, however, they were not identified as unique, according to the criteria set up to identify unique genes in this analysis.

Interestingly, 8 genes which are located adjacent to each other from NEA12_g03979 to NEA12_g03986 in the corresponding contig were identified as unique, except for one gene. NEA12_g03980 which showed very high expression in NEA12 (in the top 50 most highly expressed genes for NEA12) was not identified as unique since it showed some (very low) expression in NEA11 (maximum number of normalised reads 14.79 at 1d). Predicted functions of these genes are provided in Table 3. The gene encoding a clavaminate synthase-like protein is suggested to be involved in secondary metabolite biosynthesis and catalysis of the production of antibiotic compounds [123]. 

**Figure 7:**
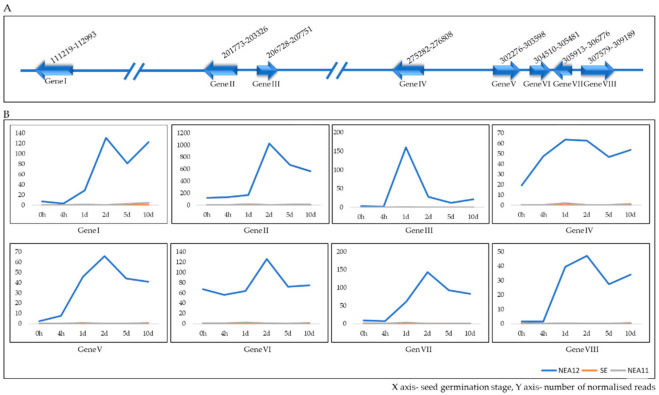
(**A**) Schematic representation of the novel potential secondary metabolite biosynthesis gene cluster (Gene I—NEA12_g03979, Gene II—NEA12_g03980, Gene III—NEA12_g03981, Gene IV—NEA12_g03982, Gene V—NEA12_g03983, Gene VI—NEA12_g03984, Gene VII—NEA12_g03985, Gene VIII—NEA12_g03986) in NEA12 pacBio contig 010_1-500000; (**B**) Expression of NEA12_g03979—NEA12_g03986 genes in NEA12 compared to SE and NEA11 at different stages of symbiotum establishment in seed germination.

Analysis of the distribution of these genes along the corresponding contig identified that they are positioned next to each other on contig 10 (from 111219-309189 bp) of the NEA12 genome (Figure 7A). With regards to their expression patterns according to fuzzy clustering, these genes belong to clusters 5, 9, and 11 (Table 3). Only NEA12_g03984 did not meet the criteria for cluster analysis. However, as shown in Figure 7B, NEA12_g03984 showed an expression pattern similar to the expression profile of cluster 9 genes. Interestingly, cluster 5 includes genes coding for both epoxy-janthitrem and lolitrem B biosynthesis, cluster 9 includes genes coding for epoxy-janthitrem biosynthesis and cluster 11 includes genes coding for lolitrem B biosynthesis in NEA12. Furthermore, as explained in the above section, the expression pattern of cluster 5 genes is common among ergovaline biosynthesis genes from both SE and NEA11. Genes in eukaryotic genomes are mostly organised in physical clusters, for example, they are located close to each other on chromosomes and clustered genes are co-expressed [115]. Consistent with the observations of this study, genes for synthesis of secondary metabolites are often arranged in clusters in fungal genomes and are often co-ordinately regulated [46,115]. The arrangement of these 8 genes (NEA12_g03979 to NEA12_g03986) along the genome and similar expression patterns together with their predicted functions indicate it is likely that this gene cluster is associated with secondary metabolism.

Another interesting finding of this analysis is identification of an NRPS, NEA12_g00506, unique to NEA12. Blast analysis identified the closest match is *Beauveria bassiana* (entomopathogenic fungus) ARSEF 2860 nonribosomal peptide synthase (GenBank accession: XM_008596907.1). 

In this analysis, genes unique to each of the three strains examined were identified based on the number of reads mapped to genes at time points of analysis and also the criteria used for selection. Therefore, it is possible that still more unique genes have not been captured in this particular analysis. Finally, the possibility of unique genes, that are lowly expressed or not expressed at the time points examined in planta, is not ruled out.

## 4. Conclusions

This is the first study to examine the genome-wide expression profiles of genes of different asexual *Epichloë* endophyte strains during the early stages of symbiotum establishment. We used a transcriptome-based approach to characterise the perennial ryegrass-endophyte symbiosis at six different stages from seed imbibition to seedling growth up to 10 days. Genes with very high expression that are present in SE, NEA11, and NEA12, but have not yet been characterised in *Epichloë* species in detail were identified. At least some of these genes are highly likely to play major roles in the endophyte-grass symbiosis as they are present in high abundance in different endophyte strains belonging to different taxonomic groups and are therefore candidates for future functional analysis.

A whole view on global gene expression changes in endophytes at different stages of symbiotum establishment that are consistent with the developmental processes of seedling development and symbiotic interaction was observed in enrichment analysis of DEGs. Gene cluster analysis provides useful insight into the expression patterns of pathway genes involved in the biosynthesis of secondary metabolites. As expression of such genes forms a consistent pattern, this analysis has proven useful for identification of genes or gene clusters responsible for production of new secondary metabolites. 

This study identified novel genes which may be beneficial for the symbiotum, in particular, novel candidate genes for secondary metabolism in NEA12 and SE. Understanding individual endophyte strain traits in more detail will enable better utilization of endophytes by avoiding animal health problems while maintaining positive impacts on pastural agriculture. For example, NEA12 is a novel endophyte strain with many favourable characteristics such as strong antifungal activity, and lack of production of the secondary metabolites known to be toxic to grazing mammals. However, there are many unidentified compounds produced by these endophytes which have implications for animal health and welfare, insect control, and bioprotection. Therefore, further studying the new genes and gene clusters identified in this analysis is important for better understanding the biochemistry of these endophytes. 

In summary, this study further highlights the benefit of harbouring an endophyte to the host, the importance of broadening our understanding of endophytes, and lays the foundation for many future studies on molecular mechanisms underlying host–endophyte symbiosis.

## Figures and Tables

**Table 1:** Properties of selected asexual *Epichloë* endophytes used in transcriptome analysis of symbioses.

Endophyte Strain	SE	NEA11	NEA12
Taxon ^a^	*Lp*TG-1	*Lp*TG-2	*Lp*TG-3
Ploidy level ^b^	haploid	heteroploid	haploid
Alkaloid biosynthesis profile ^c^	peramine lolitrem B ergovaline	peramine ergovaline	epoxy-janthitrems
Growth rate ^d^	Moderate	Fast	Slow

^a,^^b^ [48]; ^c^ [48,49]; ^d^ [47].

**Table 2:** The most abundant genes common to SE, NEA11, and NEA12.

Sequence Name		Sequence Description	Function in Fungi/ *Epichloë*
SE and NEA11	NEA12		
Ef_C09_g12381	NEA12_g08100	HSP 30	Conservation of energy in cells by inhibiting ATPase during stress conditions [63].
Ef_C09_g08349	NEA12_g01945	HSP 101	Involves in heat tolerance [70].
Ef_C09_g12810	NEA12_g01905	catalase A	Decomposes hydrogen peroxide to provide defence against oxidative stress and thereby provides a fitness advantage to pathogenic fungi in the presence of stress [41,71,72].
Ef_C09_g08943	NEA12_g03844	GPI anchored serine-rich protein	Play crucial roles in various plant–fungus interaction processes, including attachment of hyphae to surfaces, cell wall integrity and modification, virulence and degradation of host tissues [46,67,73].
Ef_C09_g11992	NEA12_g01900	grass induced protein	Suggested to play an important role in symbiosis, since it is present in high abundance in a wide range of *Epichloë*-grass associations examined thus far [34,44,74].
Ef_C09_g10329	NEA12_g04886	translation elongation factor 1-alpha	The main role is in translation. TefA is also involved in signal transduction, virus infection, nuclear export of proteins, mitochondrial tRNA import, virulence, adhesion, invasion and regulation of the immune system [69,75].
Ef_C09_g00763	NEA12_g07612	glycoside hydrolase family 10 protein	Plant cell wall degrading CAZYmes, involved in polysaccharide degradation, are particularly important for fungal pathogens due to their involvement during penetration and successful infection of their hosts [76]. Furthermore, carbohydrates released from plant cell walls are a source of nutrition for the growth of fungi [76].
Ef_C09_g02703	NEA12_g01373	glycoside hydrolase, superfamily	
Ef_C09_g10132	NEA12_g03401	glycosyltransferase family 90 protein	
Ef_C09_g07063	NEA12_g05681	endochitinase B1	
Ef_C09_g07193/Et_E8_g4372	NEA12_g00608	phosphoketolase	The phosphoketolase pathway plays an important role in central carbon metabolism of fungi and has been identified as required for full virulence of some pathogenic fungi [77,78].
Ef_C09_g11163	NEA12_g02369	hypothetical protein MAM_00430	Not characterised in detail.
Ef_C09_g11597	NEA12_g01730	cell surface protein, putative	Not characterised in detail.

**Table 3:** Predicted functions and cluster numbers of a putative novel biosynthetic gene cluster in NEA12.

Sequence Name	Sequence Description	Cluster Number *
NEA12_g03979	cystathionine gamma-synthase	5
NEA12_g03980	clavaminate synthase-like protein	5
NEA12_g03981	HpcH/HpaI aldolase/citrate lyase family protein	11
NEA12_g03982	putative efflux pump antibiotic resistance protein	9
NEA12_g03983	aspartate aminotransferase	5
NEA12_g03984	flavin-nucleotide-binding protein	
NEA12_g03985	7alpha-cephem-methoxylase P8 chain related protein	5
NEA12_g03986	putative D-aminoacylase	5

* Cluster numbers determined by fuzzy clustering shown in Figure 6.

## Data Availability

Not applicable.

## Supplementary Materials

The following are available online at https://www.mdpi.com/article/10.3390/microorganisms9050991/s1, Figure S1: MDS plots for the biological replicates of sequencing libraries of (A) SE, (B) NEA11 and (C) NEA12 at six different time points of symbiotum establishment. Table S1: BLAST hits of top 50 most abundantly expressed endophyte genes for SE, NEA11 and NEA12. Figure S2: Gene Ontology (GO) functional annotation of upregulated genes of SE at six time points of symbiotum establishment. Table S2: Enriched GO terms (Biological Processes) of the genes upregulated at different time points for SE, NEA11 and NEA12. Figure S3: Gene expression profiles of known alkaloid biosynthesis genes. Figure S4: Clustering analysis of DEGs during early stages of symbiotum establishment determined by Mfuzz for (A) SE, (B) NEA11 and (C) NEA12. Table S3: Top BLAST hits of genes identified as unique to SE from pairwise comparison of SE and NEA12. Table S4: Top BLAST hits of genes identified as unique to NEA11. Table S5: Top BLAST hits of genes identified as unique to NEA12.
