# Supplementary material for: Global Changes in Asexual Epichloë Transcriptomes during the Early Stages, from Seed to Seedling, of Symbiotum Establishment
Source: Microorganisms. 2021 May 4;9(5):991. doi: 10.3390/microorganisms9050991 (PMC8147782; doi:10.3390/microorganisms9050991)
Supplement: Supplementary file 1 [file microorganisms-09-00991-s001.zip › microorganisms-1187855-SI/Supplementary files/Figure S2.pdf]

Figure S2: Gene Ontology (GO) functional annotation of differentially expressed (upregulated) genes (DEGs) of SE at six time points of symbiotum establishment.

GO annotation was used to identify the functional categories of upregulated genes of SE based on generic terms at level 3 using the Blast2go software. Upregulated genes were categorized into 44 functional groups based on biological process (18), cellular component (17) and molecular function (9) - based on sequence homology. The annotations of gene sequences, for which GO categories could be assigned, were most frequently associated with biological processes followed by cellular components and molecular functions. Genes involved in “organic substance metabolic process”, “cellular metabolic process” and “primary metabolic process” were always predominant in the biological process category. The cellular components category was dominated by “intracellular”, “intracellular part” and “intracellular organelle” at all time points except 0h at which “intracellular”, “intracellular part” and “intrinsic component of membrane” were dominant. Moreover, a significant proportion of the genes were involved in “ion binding”, “organic cyclic compound binding” and “heterocyclic compound binding” at all time points in the category of molecular function.

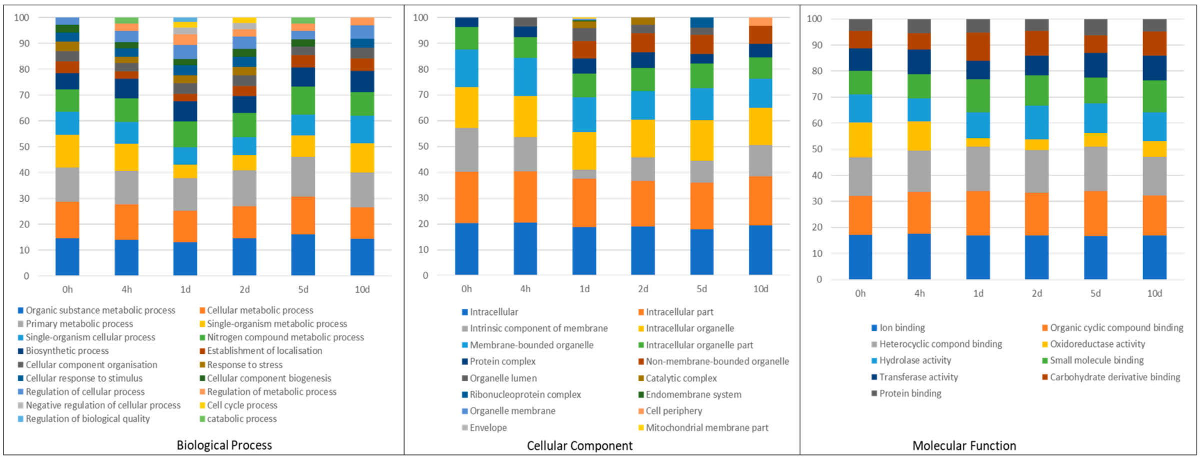

X axis- seed germination stage, Y axis- percentage of genes

Figure S2: Gene Ontology (GO) functional annotation of upregulated genes in SE at 6 time points of symbiotum establishment.
