# Supplementary material for: Global Changes in Asexual Epichloë Transcriptomes during the Early Stages, from Seed to Seedling, of Symbiotum Establishment
Source: Microorganisms. 2021 May 4;9(5):991. doi: 10.3390/microorganisms9050991 (PMC8147782; doi:10.3390/microorganisms9050991)

Figure S3: Gene expression profiles of known alkaloid biosynthesis genes

The *perA* gene, which encodes peramine, was downregulated until 1d in SE and NEA12, 2d in NEA11 and upregulated thereafter.

In both SE and NEA11, all genes required for ergot alkaloid biosynthesis were upregulated in the last 4 time points (1d, 2d, 5d and 10d) compared to the first two time points (0h and 4h), except for *easF* (0h, 1d, 10d), *easD* (1d), *easH* (1d) in SE and *easF* (0h, 10d) in NEA11. NEA12 does not contain genes for ergot alkaloid biosynthesis.

All the genes required for lolitrem B biosynthesis except *ltmB* (0h, 4h), *ltmC* (0h) and *ltmK* (4h), were downregulated until 2d in SE. At 5d, *ltmC*, *ltmK*, *ltmM*, *ltmP*, *ltmQ*, *ltmG* were upregulated while the other genes were downregulated and at 10d, all genes were upregulated. In NEA11, all the genes required for lolitrem B biosynthesis, except *ltmB* (1d), were downregulated until 2d and upregulated at 5d and 10d. For NEA12 symbiota, all the genes required for lolitrem B/epoxy-janthitrem biosynthesis were down regulated until 4h and upregulated from 1d except for *ltmF*, *ltmP* and *ltmQ*. No expression of *ltmE* and *ltmJ* genes were observed for NEA11 and NEA12, as expected.

In NEA12 symbiota, genes for epoxy-janthitrem biosynthesis were downregulated at the beginning of seed germination and upregulated later. The genes *jtmO*, *jtmD* and *PP02* are downregulated at 0h and 4h and upregulated at 1d, 2d, 5d and 10d. *PP01* is downregulated only at 0h and upregulated at the next 5 time points (4h, 1d, 2d, 5d and 10d).

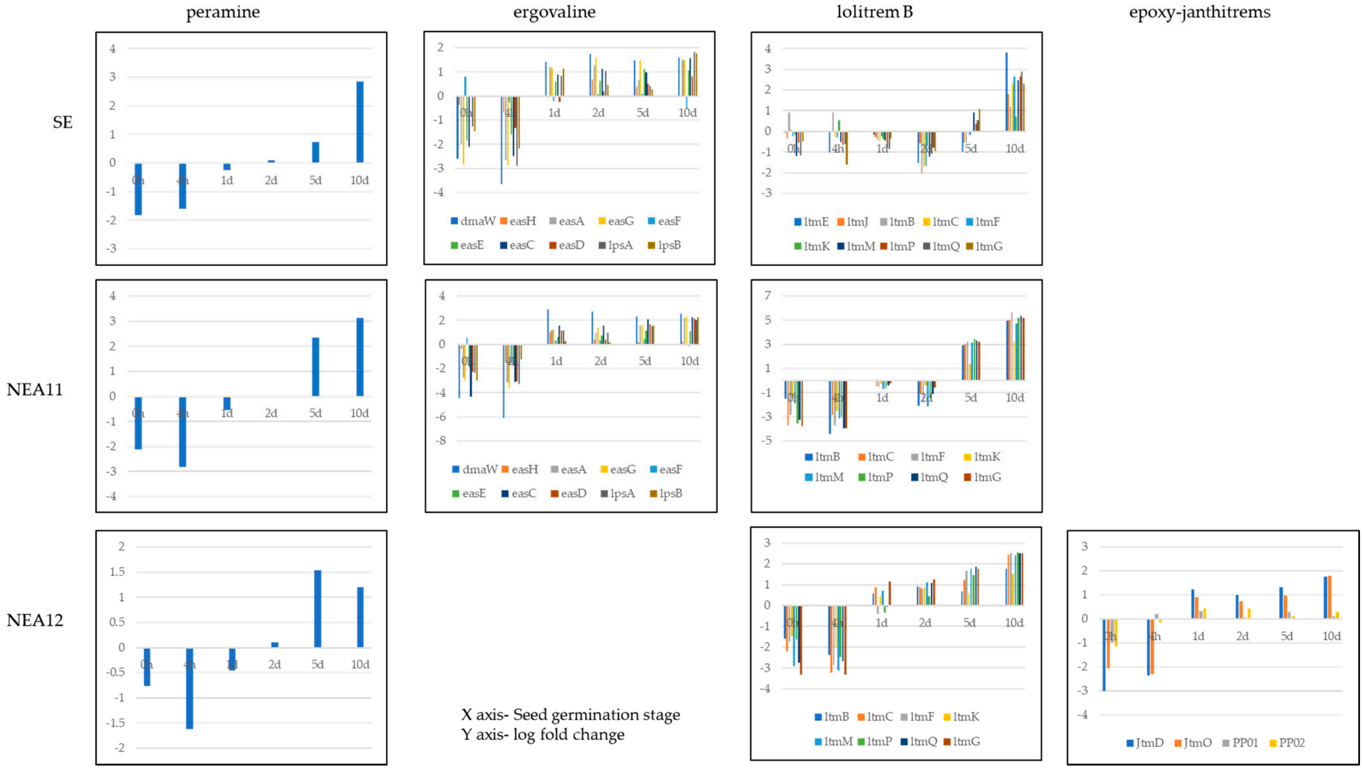

Supplement: Supplementary file 1 [file microorganisms-09-00991-s001.zip › microorganisms-1187855-SI/Supplementary files/Figure S3.pdf]
