# Supplementary material for: Global Changes in Asexual Epichloë Transcriptomes during the Early Stages, from Seed to Seedling, of Symbiotum Establishment
Source: Microorganisms. 2021 May 4;9(5):991. doi: 10.3390/microorganisms9050991 (PMC8147782; doi:10.3390/microorganisms9050991)
Supplement: Supplementary file 1 [file microorganisms-09-00991-s001.zip › microorganisms-1187855-SI/Supplementary files/Figure S4.pptx]

## Slide 1
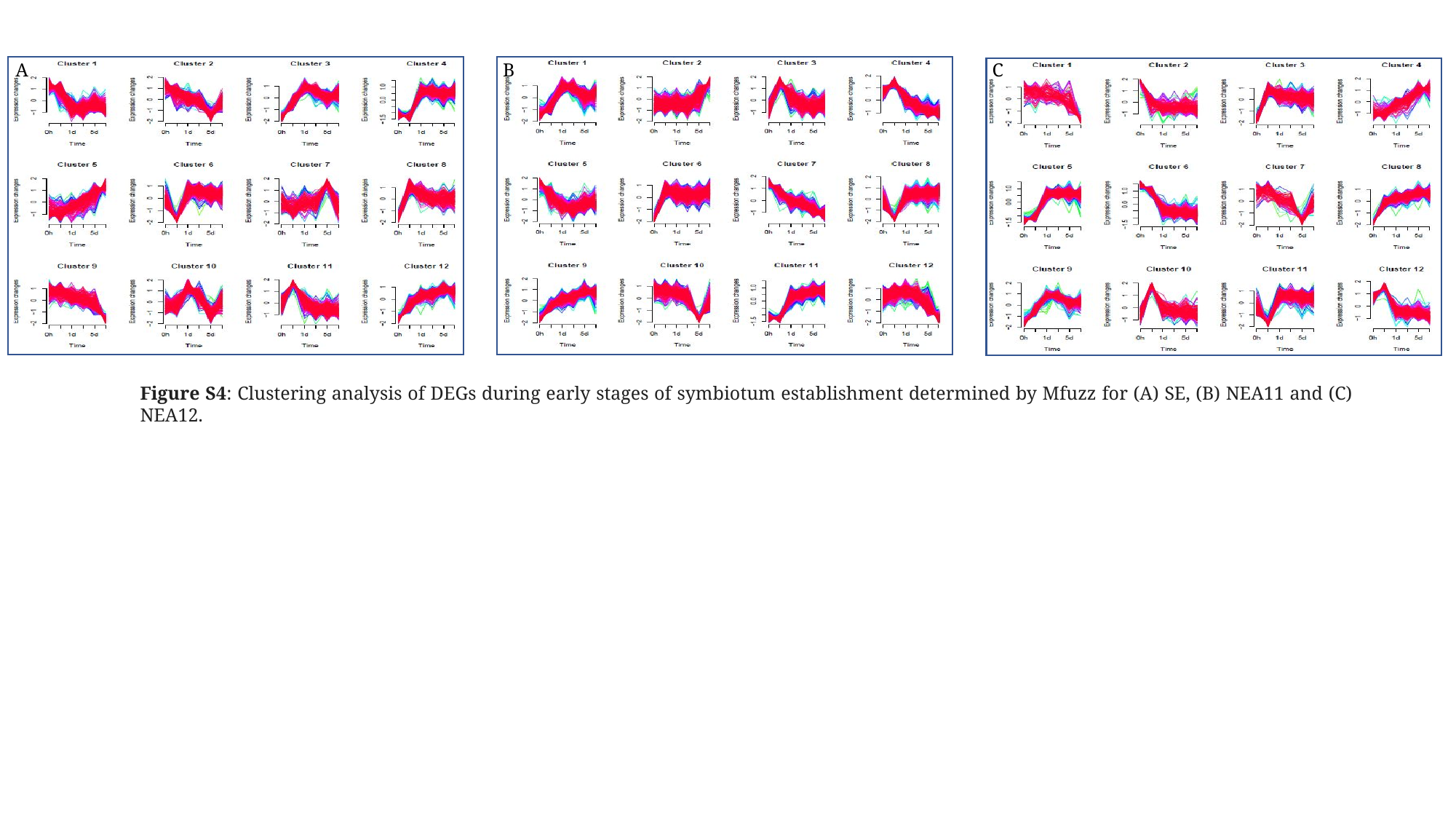

A
C
B
Figure S4: Clustering analysis of DEGs during early stages of symbiotum establishment determined by Mfuzz for (A) SE, (B) NEA11 and (C) NEA12.
